# Initial treatment patterns of primary membranoproliferative glomerulonephritis in Japan (2017–2021): an updated analysis based on nationwide personal clinical records

**DOI:** 10.1007/s10157-026-02828-7

**Published:** 2026-02-15

**Authors:** Naoki Nakagawa, Keiju Hiromura, Yoshitaka Isaka

**Affiliations:** 1 Division of Cardiology and Nephrology, Department of Internal Medicine, Asahikawa Medical University, 2-1-1-1 Midorigaoka-Higashi, Asahikawa, Japan; 2 Department of Nephrology and Rheumatology, Gunma University Graduate School of Medicine, Maebashi, Japan; 3 Department of Nephrology, The University of Osaka Graduate School of Medicine, Suita, Japan

**Keywords:** Clinical characteristics, Membranoproliferative glomerulonephritis, Nephrotic syndrome, Registry

## Abstract

**Background:**

Primary membranoproliferative glomerulonephritis (MPGN) is a rare progressive kidney disease that often leads to end-stage kidney disease. Our previous nationwide registry study (Report 1, 2015–2018) characterized initial demographics and treatment patterns. In this study (Report 2), we aimed to update these findings, focusing on newly registered cases (2017–2021).

**Methods:**

Personal clinical records of patients with primary MPGN between 2017 and 2021 were obtained from the national registry organized by the Japanese Ministry of Health, Labour and Welfare. Characteristics of primary MPGN throughout Japan were investigated.

**Results:**

A total of 210 patients (median age, 49 years; male, 51%) with newly registered primary MPGN were identified. Nephrotic syndrome was present in 83.8% of patients at enrollment. Initial treatment frequently included corticosteroids (63.8%), with a modest increase in intravenous methylprednisolone pulse therapy (41.4%) compared with that in Report 1. The incidence of hemodialysis was 7.1%. Compared with those in Report 1, the demographic patterns were similar; however, nephrotic presentations were more common. Cyclosporine and mizoribine usage were significantly higher in the pediatric group (< 18 years, *n* = 44) compared to the corresponding usage in the older adult group (≥ 65 years, *n* = 75). The mean dosage of oral prednisolone and other immunosuppressants during initial treatment did not differ among the four age groups.

**Conclusion:**

Compared with the earlier report (Report 1), patients with newly registered primary MPGN presented with nephrotic syndrome more often, highlighting the continued risk of poor prognosis and the need for more refined therapeutic approaches.

**Supplementary Information:**

The online version contains supplementary material available at 10.1007/s10157-026-02828-7.

## Introduction

Primary membranoproliferative glomerulonephritis (MPGN) is a rare immune complex- and complement-mediated pattern of injury characterized by mesangial cell proliferation and capillary wall remodeling, leading to chronic kidney injury and potential progression to end-stage kidney disease [1–3]. Membranoproliferative lesions have traditionally been classified based on the location of deposits observed on electron microscopy: Type I MPGN (MPGN I), characterized by subendothelial and mesangial electron-dense deposits containing both immunoglobulin and C3; Type II MPGN (MPGN II, known as dense deposit disease [DDD]), characterized by electron-dense intramembranous deposits predominantly composed of complement; and Type III MPGN, characterized by both subepithelial and subendothelial deposits [4]. Although this traditional classification does not reflect underlying disease pathogenesis, patients with MPGN who present with severe conditions are eligible for medical expense aid under the intractable disease program of the Japanese Ministry of Health, Labour and Welfare, which is based on personal clinical records and employs the traditional classification system. Our previous nationwide registry analysis of personal clinical records, which included all registered cases such as new registrations and updates (Report 1, 2015–2018) [5], provided the first comprehensive demographic and treatment overview of Japanese patients with primary MPGN and identified a bimodal age distribution, near-equal sex ratio, and high corticosteroid usage [5]. Nevertheless, evolving diagnostic awareness, therapeutic strategies, and registry completeness necessitate an updated analysis.

Primary MPGN encompasses C3 glomerulopathy [1]. Recently, novel complement inhibitors for C3 glomerulopathy have garnered substantial attention [6, 7], one of which is currently available in Japan [8]. Thus, elucidating the current clinical landscape and treatment practices for MPGN in Japan is important. In this study (Report 2), we aimed to examine demographic and clinical presentation patterns, initial treatment strategies, and early dialysis initiation rates in this population using only newly registered cases of personal clinical records from April 2017 to June 2021. These data are essential for understanding the current disease burden and for informing clinical decision-making.

## Materials and methods

### Overview of personal clinical records

This cross-sectional study used data from personal clinical records in the “National Database of Designated Incurable Diseases of Japan,” a nationwide administrative database of public expenditure for refractory diseases (including primary MPGN) throughout Japan that is maintained by the Japanese Ministry of Health, Labour and Welfare [9, 10]. The records prospectively and annually collected demographic data (age and sex), chronic kidney disease (CKD) classification based on glomerular filtration rate (GFR) and proteinuria (A1, < 0.15; A2, 0.15–0.49; A3, ≥ 0.5 g/day or g/gCr), medication use (presently used and maximum dosage of the present treatment within 6 months), and initiation of renal replacement therapy. Early dialysis initiation was defined as starting dialysis within 1 year before registration. The data were registered only following a review by certified nephrologists; however, the database did not contain survival data, including death. Our findings are original and differ from the statistics produced or published by the Japanese Ministry of Health, Labour and Welfare [9, 10]. The Ministry’s statistics are not generally available to the public.

Personal clinical records are registered through two methods, namely, new registrations and updates. New registrations are typically submitted when a new case is diagnosed and the initial treatment begins. In this study, we analyzed newly registered primary MPGN cases to determine the current initial treatment patterns in Japan (https://www.nanbyou.or.jp/entry/4423, in Japanese). Severity classification was defined as follows:

- Children (aged < 18 years) who meet the following criteria: a or b.
- Diagnosis confirmed by pathological examination and treatment using one or more of the following: steroids, immunosuppressants, biologics, anticoagulants, antiplatelet drugs, albumin preparations, or antihypertensive drugs.
- Patients who have undergone kidney transplantation.
- Adults who meet any of the following criteria:
- Patients located in the red area (very high-risk) of the CKD severity classification heat map.
- Patients who meet the criteria for nephrotic syndrome.
- Patients who have not achieved remission despite immunosuppressive treatment (including steroid treatment) or who have persistent hypocomplementemia.

### Study population

In the present study, personal clinical records of newly registered patients with type I or III primary MPGN from April 2017 to June 2021 were used. Primary MPGN was diagnosed based on renal biopsy findings in the absence of type II MPGN (dense deposit disease) and any secondary MPGN [3], including autoimmune diseases (lupus nephritis, immunoglobulin A vasculitis, and other types of vasculitis), paraproteinemia (amyloidosis, cryoglobulin, heavy-chain deposition, and light-chain deposition), infectious diseases (streptococcal and staphylococcal infections, hepatitis B and C infections, human immunodeficiency virus infection, parvovirus B19 infection, bacterial endocarditis, and shunt nephritis), tumors (malignant lymphoma and leukemia), genetic diseases associated with complement dysfunction, or liver diseases (liver cirrhosis and antitrypsin deficiency). Nephrotic syndrome was diagnosed based on massive proteinuria (≥ 3.5 g/day) and hypoalbuminemia (serum albumin level ≤ 3.0 g/dL) [11]. In 2015, primary MPGN was recognized as an intractable disease in Japan (designated as intractable disease number 223; https://www.nanbyou.or.jp/entry/4423, in Japanese). The clinical records of patients with primary MPGN included survey items regarding renal replacement therapy and pathological findings of a modified Japanese classification [12] (Table S1). However, the immunostaining findings were not collected in this system.

### Data curation

For statistical analysis, the data were processed and refined in advance to render them suitable for analysis. The presence of a duplicate ID indicated duplicate data, and only one record was retained. Erroneous data (e.g., values that were entered despite the absence of tests performed) were included after likely values were assigned as alternatives. These values were determined by referring to the basic statistics of the data.

### Characteristics and management of primary MPGN

Demographics (age at onset and sex), CKD classification based on GFR and proteinuria, and management of primary MPGN were determined in this registry. Information on the use and mean dosage of initial steroids and immunosuppressive drugs was obtained. Steroid resistance was defined as never achieving complete remission despite immunosuppressive treatment, including steroid therapy, according to the definition outlined in the Japanese guidelines for nephrotic syndrome [13]. Patients with MPGN were divided into four age groups: pediatric group (< 18 years), adolescent and young adult group (18–39 years), middle-aged adult group (40–64 years), and older adult group (≥ 65 years). Clinical parameters were compared among these four age groups according to our previous report [5].

### Statistical analysis

Quantitative variables were expressed as means (standard deviations) for normally distributed data or as medians (interquartile ranges). Qualitative variables were presented as frequencies (percentages). Differences between groups were compared using the chi-square test or Fisher’s exact test for categorical variables and the non-parametric Kruskal–Wallis test for continuous variables. All data were analyzed using IBM SPSS version 26.0 (IBM Corp., Armonk, NY, USA), with statistically significant difference defined as *P* < 0.05.

## Results

### Distribution and sex differences of primary MPGN according to age at onset in the national registry of personal clinical records

**Fig. 1:**
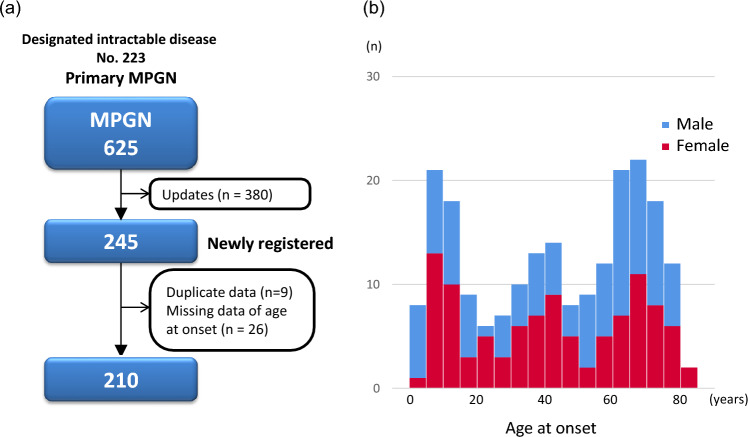
**a** Flow diagram of patient selection from the national registry of personal clinical records of primary membranoproliferative glomerulonephritis (MPGN). **b** Distribution of glomerulopathies in the national registry of primary MPGN according to sex and age (in years) at onset

A total of 625 cases of primary MPGN were enrolled as primary MPGN (designated as intractable disease number 223) in the nationwide registry of personal clinical records from April 2017 to June 2021. Of these 625 cases, 245 were newly registered cases (Fig. 1). After removing duplicate (*n* = 9) and missing data of age at onset (*n* = 26), 210 patients with primary MPGN were included in the analysis (Fig. 1a). The median age at onset was 49 years, with an early peak at 5–14 years and a later peak at 60–69 years (Fig. 1b). Male individuals accounted for 51% of this analysis, which is consistent with the 1:1 sex ratio observed in Report 1. A female predominance was noted in young and middle-aged individuals, whereas a male predominance was observed in older adults (Fig. 1b). Nephrotic syndrome was present in 83.8% of patients, which is higher than 77.1% reported in Report 1. Of 210 patients with primary MPGN, 15 (7.1%) underwent hemodialysis for end-stage kidney disease (Table 1). The most common G and A stages of the CKD risk classification were G3b (22.9%) and A3 (90.5%), respectively (Table 1).

**Table 1:** Demographics of newly registered primary membranoproliferative glomerulonephritis in the national registry of personal clinical records

	All
*N*	210
Age at onset (years)	49 (21, 70)
Male, *n* (%)	107 (51.0%)
CKD G stage	
G1	35 (16.7%)
G2	27 (12.9%)
G3a	34 (16.2%)
G3b	48 (22.9%)
G4	45 (21.4%)
G5	21 (10.0%)
CKD A stage	
A1	10 (4.8%)
A2	10 (4.8%)
A3	190 (90.5%)
Nephrotic syndrome	176 (83.8%)
Initial treatment	
Oral prednisolone	134 (63.8%)
IV methylprednisolone	87 (41.4%)
Cyclosporine	49 (23.3%)
Mizoribine	37 (17.6%)
Cyclophosphamide	8 (3.8%)
Mycophenolate mofetil	8 (3.8%)
Rituximab	7 (3.3%)
Tacrolimus	6 (2.9%)
Azathioprine	4 (1.9%)
Dosage of initial treatment	
Oral prednisolone (mg/day)	42.5 ± 10.5
IV methylprednisolone (mg/day)	655.2 ± 235.8
Cyclosporine (mg/day)	106.5 ± 38.0
Mizoribine (mg/day)	152.7 ± 91.4
Cyclophosphamide (mg/day)	90.6 ± 70.6
Mycophenolate mofetil (mg/day)	1,031.3 ± 339.1
Rituximab (mg/day)	500.0 ± 0.0
Tacrolimus (mg/day)	1.9 ± 0.8
Azathioprine (mg/day)	56.3 ± 31.5
Initiation of hemodialysis	15 (7.1%)

Data are expressed as means ± SD, medians (interquartile ranges), or numbers (percentages)

*CKD* chronic kidney disease

Oral prednisolone and intravenous methylprednisolone pulse therapy were used in 63.8% and 41.4%, respectively, followed by cyclosporine (23.3%) and mizoribine (17.6%) (Table 1) during the initial treatment. The mean oral prednisolone dose was 42.5 ± 10.5 mg/day during the initial treatment. Most immunosuppressive drugs were administered with oral prednisolone (Table S2).

### Comparison among three age groups of patients with primary MPGN

**Fig. 2:**
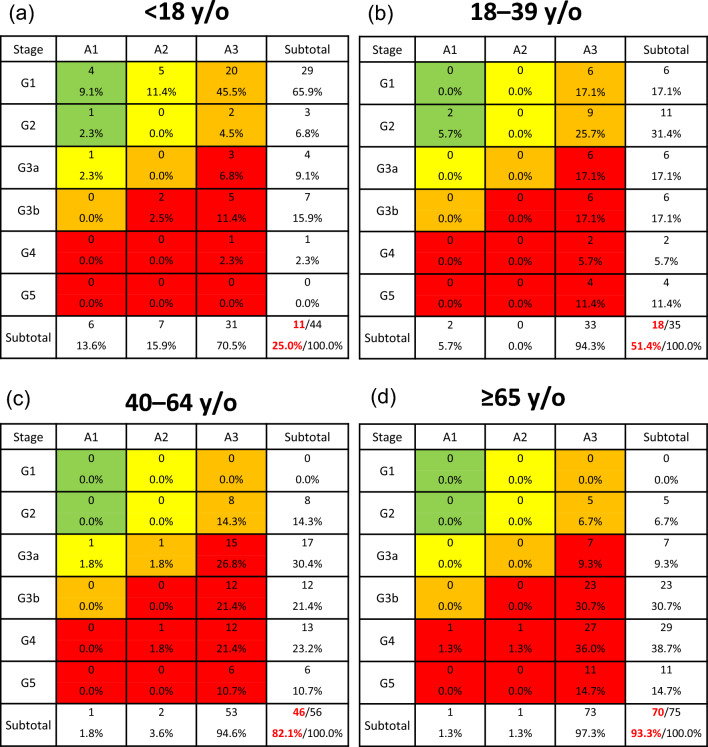
Risk classification of chronic kidney disease (CKD) in three age groups of patients with primary membranoproliferative glomerulonephritis: **a** adolescent and young adult group (≤ 39 years), **b** middle-aged adult group (40–64 years), and **c** older adult group (≥ 65 years). Green: low risk (no other markers of kidney disease, no CKD); yellow: moderately increased risk; orange: high risk; red: very high risk

Patients were divided into four age groups: pediatric group (< 18 years), adolescent and young adult group (18–39 years), middle-aged adult group (40–64 years), and older adult group (≥ 65 years) (Table 2), because pediatric patients are typically managed in pediatric clinical settings. The frequency of nephrotic syndrome did not differ significantly among these four age groups. According to the CKD risk classification, very high-risk (red zone) patients accounted for 25% of pediatric, 51.4% of adolescents and young adults, 82.1% of middle-aged adults, and 93.3% of older adults (*P* for trend < 0.001) (Fig. 2). The use of oral cyclosporine and mizoribine was significantly higher in the pediatric group than in the older adult group, whereas the mean dosages of these drugs during initial treatment showed no differences among the four age groups. Hemodialysis was initiated in 13.5% of older adults compared with 0.0%, 5.9%, and 5.5% of pediatric, adolescent and young adults, and middle-aged adults, respectively.

**Table 2:** Demographics of cases with primary membranoproliferative glomerulonephritis according to the age at onset

	< 18 years	18–39 years	40–64 years	≥ 65 years	*P* value
*N*	44	35	56	75	
Age at onset (years old)	13 (10, 16)	28 (21, 36)	50 (45, 58)	73 (69, 78)	< 0.001^a,b,c,d,e,f^
Male	22 (50.0%)	16 (45.7%)	28 (50.0%)	41 (54.7%)	0.842
Nephrotic syndrome	28 (63.6%)	33 (94.3%)	47 (83.9%)	68 (90.7%)	< 0.001^a,b,c^
Present treatment					
Oral prednisolone	29 (65.9%)	24 (68.6%)	33 (58.9%)	48 (64.0%)	0.801
IV methylprednisolone	21 (47.7%)	19 (54.3%)	21 (37.5%)	26 (34.7%)	0.183
Cyclosporine	16 (36.4%)	16 (45.7%)	9 (16.1%)	8 (10.7%)	< 0.001^c,d,e^
Tacrolimus	1 (2.3%)	4 (11.4%)	0 (0.0%)	1 (1.3%)	0.009^d,e^
Cyclophosphamide	2 (4.5%)	0 (0.0%)	1 (1.8%)	5 (6.7%)	0.292
Mizoribine	16 (36.4%)	8 (22.9%)	6 (10.7%)	7 (9.3%)	0.001^b,c^
Mycophenolate mofetil	3 (6.8%)	3 (8.6%)	2 (3.6%)	0 (0.0%)	0.102
Azathioprine	1 (2.3%)	1 (2.9%)	1 (1.8%)	1 (1.3%)	0.953
Rituximab	1 (2.3%)	4 (11.4%)	1 (1.8%)	1 (1.3%)	0.035^e^
Dosage of present treatment					
Oral prednisolone (mg/day)	41.4 ± 13.0	45.0 ± 12.0	44.6 ± 9.9	40.3 ± 8.0	0.216
IV methylprednisolone (mg/day)	773.8 ± 248.8	657.9 ± 238.8	619.1 ± 218.2	586.5 ± 211.5	0.052
Cyclosporine (mg/day)	102.3 ± 32.0	107.8 ± 37.3	127.8 ± 49.1	87.5 ± 29.9	0.075
Tacrolimus (mg/day)	2.5	1.75 ± 1.0	–	2.0	1.000
Cyclophosphamide (mg/day)	50.0 ± 0.0	–	125.0	100.0 ± 86.6	0.307
Mizoribine (mg/day)	175.0 ± 121.1	134.4 ± 51.6	147.2 ± 8.3	114.3 ± 24.4	0.331
Mycophenolate mofetil (mg/day)	1333.3 ± 288.7	916.7 ± 144.3	750.0 ± 353.6	–	0.211
Azathioprine (mg/day)	100.0	50.0	25.0	50.0	0.325
Rituximab (mg/month)	500.0	500.0	500.0	500.0	1.000
Initiation of hemodialysis	0 (0.0%)	2 (5.9%)	3 (5.5%)	10 (13.5%)	0.044^c^

Data are expressed as means ± SD, medians (interquartile ranges), or numbers (percentages)

^a^*P* < 0.05, < 18 years vs. 18–39 years

^b^*P* < 0.05, < 18 years vs. 40–64 years

^c^*P* < 0.05, < 18 years vs. ≥ 65 years

^d^*P* < 0.05, 18–39 years vs. 40–64 years

^e^*P* < 0.05, 18–39 years vs. ≥ 65 years

^f^*P* < 0.05, 40–64 years vs. ≥ 65 years. Kruskal–Wallis tests with Bonferroni-corrected *P* values

### Pathological findings in patients with primary MPGN

Pathological findings were identified in 208 out of 210 primary MPGN cases. The most common finding was a diffuse and global lobular appearance with double contours of the glomerular basement membrane (32.2%), followed by moderate mesangial proliferation (25.9%) and moderate diffuse and global double contours of the glomerular basement membrane (25.0%) (Table S3).

## Discussion

To our knowledge, this is the first study to describe the clinical features and real-world management of patients with newly registered primary MPGN throughout Japan, including both the use and mean dosage of immunosuppressants during initial treatment dividing four age groups, according to a national database of > 200 patients.

The characteristic age distribution was consistent across both Report 1 (2015–2018; newly registered and updates) and Report 2 (2017–2021; only newly registered) of the present study, exhibiting a bimodal pattern, with peaks in prevalence among adolescents and older adults. The sex ratio remained stable at approximately 1:1 in both reports. Furthermore, the rate of initial corticosteroid use was comparable, observed in approximately 60–70% of patients in both study periods. Corticosteroids were prescribed to approximately 63.8% of the patients, similar to that in Report 1 (70.5%). However, a significant difference in the prevalence of nephrotic syndrome (83.8%) was observed. The use of intravenous methylprednisolone pulse therapy increased (from 6.2% to 41.4%), whereas the use of other immunosuppressants remained infrequent. This suggests that (1) Report 2 had many cases of early nephrotic syndrome that were treated intensively, and (2) Report 1 had updated cases whose nephrotic syndrome might have remitted following treatment by the time of data collection. Additionally, Report 2 uniquely identified that 7.1% of all newly registered cases, including young adult patients, required renal replacement therapy within 1 year of enrollment, providing initial insights into early renal outcomes.

Regarding the comparison between the four age groups, pediatric (< 18 years) patients with primary MPGN received a higher prescription rate of oral cyclosporine and mizoribine than older adult patients, suggesting that immunosuppressive drugs, such as cyclosporine and mizoribine, may be used to induce long-lasting remission, sparing pediatric patients from further steroid exposure. By contrast, the mean dosages of oral prednisolone and other immunosuppressants during initial treatment did not differ among the four age groups because nephrotic syndrome was more common in older adults; therefore, immunosuppressive treatment was required even in older adults with primary MPGN.

This study had some limitations. First, the database does not contain data on complications (e.g., hypertension and diabetes), genetic testing, non-pharmacological therapy, and patient outcomes. Furthermore, the database did not contain data on urine protein levels, estimated GFR, or serum albumin and complement levels. Second, we demonstrated the characteristics of primary MPGN throughout Japan using a descriptive analysis. However, temporal trend analysis would provide more clinically relevant findings. Further studies using temporal trend analysis are needed to reveal longitudinal demographic changes and rates of guideline-directed medical treatment. Third, pathological findings were only available for approximately one-third of all cases. Fourth, we could not distinguish between immune complex-mediated MPGN and C3 nephropathy [4] owing to the absence of immunostaining findings in the personal clinical records. Fourth, Japanese pediatric cases are often registered in the “Pediatric Chronic Specified Disease” program [14] rather than in the designated incurable disease database; consequently, children are likely underrepresented in this study. Fifth, mild MPGN cases that do not meet the criteria for registration in the designated incurable disease system are likely excluded, as this registry primarily captures moderate-to-severe cases, particularly among new registrations. Therefore, the extent to which the study population represents all patients with MPGN remains uncertain. Despite these limitations, we believe that the outcomes of this study will provide an insightful overview of the clinical characteristics and features of patients with primary MPGN throughout Japan, in addition to developing optimal management strategies for these patients.

In conclusion, by analyzing a nationwide database of > 200 patients with newly registered primary MPGN in the nationwide registry of Japan, this study revealed their clinical characteristics and provided critical insights for developing optimized therapeutic strategies and refining the diagnostic classification to improve renal outcomes of this rare disease. Further investigations are required to improve therapeutic strategies against primary MPGN in Japan.

## Supplementary Information

Below is the link to the electronic supplementary material.

Supplementary file1 (DOCX 27 kb)

## Data Availability

The datasets generated and analyzed during the current study are not publicly available because the consent obtained from the participants does not cover unlimited public sharing of data.
