# Supplementary material for: Initial treatment patterns of primary membranoproliferative glomerulonephritis in Japan (2017–2021): an updated analysis based on nationwide personal clinical records
Source: Clin Exp Nephrol. 2026 Feb 15;30(4):590–6. doi: 10.1007/s10157-026-02828-7 (PMC13009048; doi:10.1007/s10157-026-02828-7)
Supplement: Supplementary file 1 — Supplementary file1 (DOCX 27 kb) [file 10157_2026_2828_MOESM1_ESM.docx]

**Initial treatment patterns of primary membranoproliferative glomerulonephritis in Japan (2017–2021): An updated analysis based on nationwide personal clinical records**

Naoki Nakagawa^1^*, Keiju Hiromura^2^, and Yoshitaka Isaka^3^

^1^Division of Cardiology and Nephrology, Department of Internal Medicine, Asahikawa Medical University, Asahikawa, Japan

^2^Department of Nephrology and Rheumatology, Gunma University Graduate School of Medicine, Maebashi, Japan

^3^Department of Nephrology, Osaka University Graduate School of Medicine, Suita, Japan.

*Corresponding author: Naoki Nakagawa, MD, PhD, Division of Cardiology and Nephrology, Department of Internal Medicine, Asahikawa Medical University, 2-1-1-1 Midorigaoka-higashi, Asahikawa, Japan, Phone: +81-166-68-2442, Fax: +81-166-68-2449, E-mail: [naka-nao@asahikawa-med.ac.jp](mailto:naka-nao@asahikawa-med.ac.jp) (NN)

Table S1. Survey items of pathological findings of primary membranoproliferative glomerulonephritis in the clinical personal records.

| 1. Glomerular injury pattern of membranoproliferative glomerulonephritis |
| --- |
| 1. Mesangial proliferation |
| □ 1a. slight |
| □ 1b. moderate |
| 2. Chronic and focal |
| □ focal, segmental or global mesangial proliferation and double contours of the GBM |
| 3. Acute and focal |
| □ focal, segmental or global mesangial and endocapillary proliferation with inflammatory cells (neutrophil) without double contours of GBM |
| 4. Chronic and diffuse |
| □ 4a. slight segmental double contours of GBM |
| □ 4b.non-lobular, diffuse and global double contours of GBM |
| □ 4c.moderate diffuse and global double contours of GBM |
| 5. Acute and diffuse |
| □ 5a. endocapillary proliferation with inflammatory cells (neutrophil) |
| □ 5b. moderate endocapillary proliferation with subendothelial deposits |
| □ 5c. marked endocapillary proliferation with subendothelial deposits |
| 6. Lobular |
| □ diffuse and global lobular appearance with double contours of GBM with nodular mesangial sclerosis in the moderately enlarged glomeruli |
| 7. End stage |
| □ diffuse and global glomerulosclerosis |
| 1. Additional findings |
| 1. Glomerular lesion |
| A.　Global sclerotic lesion ％，Segmental sclerotic lesion ％  B.　Crescentic lesion　　　　 ％  C.　Degree of infiltration of white blood cells 1. － 2. ± 3. ＋ 4. ＋＋  D.　Degree of infiltration of foam cells　　　 1. － 2. ± 3. ＋ 4. ＋＋  E.　Degree of subendothelial deposits　　 1. － 2. ± 3. ＋ 4. ＋＋ |
| 1. Tubulointerstitial lesion |
| A.　Tubulointerstitial change in the cortex（％）　　　　　　　％  B.　Degree of foam cellularization of tubular epithelium 1.－ 2.± 3.＋ 4.＋＋ |
| 1. Vascular lesion |
| Degree of arteriosclerosis　　 1. － 2. ± 3. ＋ 4. ＋＋ |

GBM, glomerular basement membrane.Table S2. Initial treatment of oral prednisolone with other immunosuppressive drugs

|  | All |
| --- | --- |
| N | 210 |
| Initial treatment of oral PSL with other drugs |  |
| Oral PSL alone | 40 (19.0%) |
| Oral PSL + IV methylprednisolone | 39 (18.6%) |
| Oral PSL + IV methylprednisolone + Cyclosporine | 28 (13.3%) |
| Oral PSL + IV methylprednisolone + Mizoribine | 17 (8.1%) |
| Oral PSL + Cyclosporine | 12 (5.7%) |
| Oral PSL + Mizoribine | 14 (6.7%) |
| Oral PSL + Cyclophosphamide | 8 (3.8%) |
| Oral PSL + Mycophenolate mofetil | 7 (3.3%) |
| Oral PSL + Rituximab | 7 (3.3%) |
| Oral PSL + Tacrolimus | 6 (2.9%) |
| Oral PSL + Azathioprine | 4 (1.9%) |

PSL, prednisolone.

Table S3. Pathological findings of primary membranoproliferative glomerulonephritis

|  | All |
| --- | --- |
| N | 208 |
| Glomerular injury pattern |  |
| 1. Mesangial proliferation | 86 (41.3%) |
| 1a. slight | 32 (15.4%) |
| 1b. moderate | 54 (25.9%) |
| 2. Chronic and focal | 47 (22.6%) |
| 3. Acute and focal | 10 (4.8%) |
| 4. Chronic and diffuse | 110 (52.9%) |
| 4a. slight | 30 (14.4%) |
| 4b.non-lobular | 28 (13.5%) |
| 4c. moderate | 52 (25.0%) |
| 5. Acute and diffuse | 41 (19.7%) |
| 5a. endocapillary | 11 (5.2%) |
| 5b. moderate | 28 (13.5%) |
| 5c. marked | 2 (1.0%) |
| 6. Lobular | 67 (32.2%) |
| 7. End stage | 3 (1.4%) |
| Additional findings |  |
| Glomerular lesion |  |
| Global sclerotic lesion | 17.9 ± 20.6% |
| Segmental sclerotic lesion | 6.5 ± 13.6% |
| Crescentic lesion | 4.8 ± 11.5% |
| Infiltration of white blood cells* | 2.3 ± 1.0 |
| Infiltration of foam cells* | 1.6 ± 0.9 |
| Subendothelial deposits* | 2.9 ± 1.0 |
| Tubulointerstitial lesion |  |
| Tubulointerstitial change in the cortex | 21.3 ± 18.6% |
| Foam cellularization of tubular epithelium* | 1.8 ± 1.0 |
| Vascular lesion |  |
| Arteriosclerosis* | 2.1 ± 1.0 |

Data are expressed as the mean ± SD, median (interquartile range), or number (percentage).

*Score of degree: 1-4.
